# An Inexpensive, Stable, and Accurate Relative Humidity Measurement Method for Challenging Environments

**DOI:** 10.3390/s16030398

**Published:** 2016-03-18

**Authors:** Wei Zhang, Hong Ma, Simon X. Yang

**Affiliations:** School of Engineering, University of Guelph, Guelph, ON N1G 2W1, Canada; wzhang01@uoguelph.ca (W.Z.); hongma@uoguelph.ca (H.M.)

**Keywords:** drying rooms, measurement accuracy, relative humidity, psychrometric sensors

## Abstract

In this research, an improved psychrometer is developed to solve practical issues arising in the relative humidity measurement of challenging drying environments for meat manufacturing in agricultural and agri-food industries. The design in this research focused on the structure of the improved psychrometer, signal conversion, and calculation methods. The experimental results showed the effect of varying psychrometer structure on relative humidity measurement accuracy. An industrial application to dry-cured meat products demonstrated the effective performance of the improved psychrometer being used as a relative humidity measurement sensor in meat-drying rooms. In a drying environment for meat manufacturing, the achieved measurement accuracy for relative humidity using the improved psychrometer was ±0.6%. The system test results showed that the improved psychrometer can provide reliable and long-term stable relative humidity measurements with high accuracy in the drying system of meat products.

## 1. Introduction

Relative humidity is at all temperature and pressures defined as the ratio of the water vapour pressure to the saturation water vapour pressure (over water) at the gas temperature [1]. There are many methods to measure the relative humidity [2,3,4,5]. The measurement of relative humidity, however, depends on several environmental factors. Obtaining reliable, long-term stable, accurate and precise measurements of relative humidity remains a difficult practical issue. The task is particularly difficult in a dirty or tough measurement environment containing corrosive materials, gas pollution, or water in all three physical states. Despite numerous available relative humidity sensors designed for different measurement purposes, there are few sensors that can be used in a harsh environment with high measurement accuracy. However, practical applications, such as the drying system for meat manufacturing in agricultural, agri-food industries, as well as the dirty industrial environments or in technical spaces of ships (e.g., engine rooms) [6], require a relative humidity sensor that can provide stable, full-range measurements with high measurement accuracy in severe environments [7,8].

**Figure 1:**
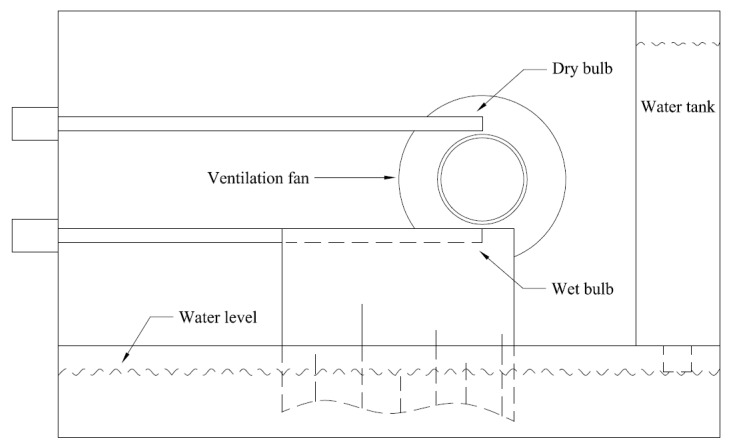
The structure of an aspirated psychrometer.

The psychrometric method of relative humidity measurement has been known for a very long time. The psychrometer measures the relative humidity based on evaporative cooling. The common structure of an aspirated psychrometer is shown in Figure 1. The device contains two temperature probes as measurement elements. In industrial applications, the temperature probe of an aspirated psychrometer can be either the thermocouple temperature sensor or the resistance thermometer. One probe is placed to measure the temperature of the ambient air, which is called the dry bulb temperature. Another probe wrapped with a wet cloth is placed close to the dry bulb to measure the cooling effect of evaporation. Its reading is called the wet bulb temperature. A fan is used for ventilation of the surrounding air. The psychrometer is an indirect method of relative humidity measurement.

A prominent advantage of a psychrometer is its adaptability to measurement environments. Compared to modern electronic relative humidity sensors such as capacitive relative humidity sensors or resistive relative humidity sensors, the psychrometer is one of the relative humidity measurement devices that can be used in a dirty environment, can withstand state changes of water, and can provide a large range of measurement values. The psychrometer is used in different measurement environment in many current applications [9,10,11,12,13].

However, as a relative humidity sensor in application system, the weakness of the psychrometer is its low measurement accuracy compared to electronic sensors [14,15,16]. The relative humidity measurement of psychrometer relies on a number of factors such as the degree of ventilation (volumetric flow rate), the diameter of the wire, the thickness and length of the water film covering the junction of the wet thermocouple, and the relative positions of the wet and dry thermocouples [17]. The psychrometer also requires skilled operators to make accurate measurements [18]. The effects of various factors such as atmospheric pressure, size of a psychrometer, ventilation conditions, or freezing of wet bulbs have been examined and support measurement theory [19]. The complexity of the wet bulb measurement environment is a limiting factor in improving its measurement accuracy. Hence, the key to increasing psychrometer measurement accuracy is to provide manufactures with specific structural design elements that ensure reliability and stability in measurements.

The wet bulb temperature should be not a thermodynamic property, but, under certain conditions it well approximates the thermodynamic wet bulb temperature [20]. The sensor must be shielded from radiation. This is a very crucial issue because a lot of researchers made use of natural wet bulb thermometers with unforeseeable consequences in terms of measurements [21]. 

A number of prior works studied the psychrometer measurement method in different environments and the effect of different factors influencing measurement accuracy. Yin *et al.* calculated the correlation coefficients of psychrometer with varying wind speeds. An aspirated psychrometer was designed and tested for broiler chicken houses [22]. The test results showed the aspirated psychrometer could work reliably in a dusty environment with minor maintenance required only every five to ten days [23]. Duuren operated four types of equipment for measuring the moisture content of air for seven months in heavy clay dryers that included the psychrometer with wet and dry bulb sensors [24].

Several researchers discuss the measurement accuracies of psychrometer. Ustymczuk and Giner examined the errors of psychrometric measurement of relative humidity and used an error propagation formula to convert temperature errors to relative humidity errors [10]. Mittal and Zhang developed a trained artificial neural network based psychrometric chart that can be used to predict psychrometric parameters in practical applications [25]. Montanini worked on a wavelength-encoded optical psychrometer for relative humidity measurement and the measurement accuracy was estimated to be within 2% relative humidity (RH) in the range close to saturation [26]. Cai *et al.* designed an intelligent dry and wet bulb relative humidity sensor, with simulation and test results demonstrating the accuracy to within 2% RH [27]. The accuracy of the digital ventilated psychrometer that was designed by Nantou is less than 2% RH [28]. The statistical results for the DYZ-1 ventilated psychrometer were shown to have a standard deviation of temperature and relative humidity of 0.23 °C and 1.7% RH [29]. The test results based on the on-line method of Bhuyan's were compared with a standard hygrometer and claimed to have a deviation of no more than 2% RH [30]. Nichols developed a relative humidity sensor utilizing the psychrometric process for poultry farming, with an expected tolerance of 1% RH for a range of several known relative humidity values [31]. Nakahama applied the Sprung formula to optimize psychrometer construction when the wind speed is above 1.5 m/s [32]. When the wind speed is in the range of 0.5 to 1.5 m/s, the Partner formula with weak wind coefficient could be used.

A psychrometer measures the relative humidity indirectly from two temperature probes. By the structure of the dry and wet bulb probes, the temperature sensing element is encased within a stainless steel tube. The sensing element is not in direct contact with the environment. The psychrometer avoids directly exposing the relative humidity sensing element to the measurement environment. This key difference allows the psychrometer to be used in difficult measurement environments. However, the relative humidity measurement process of the psychrometer is a complex physical process. Many factors can influence the measurement result such as barometric pressure, ambient temperature, velocity of the air flow, accuracy of temperature probes, location of the probes, wet cloth size and quality, *etc.* Moreover, the accuracy of conversion formula, measurement circuits, and calculation equipment are important in the psychrometer measurement process. These factors combine to affect the measurement result and limit the widespread usage of the psychrometer.

Nakahama investigated the psychrometer construction for performance testing in temperature and humidity chambers and the accuracy of humidity measurements meeting the challenge of quality engineering [32]. Nakahama states “Due to the improvement of humidity measurement technology and the development of traceability systems in recent years, we have again taken up the challenge of verifying psychrometer construction and humidity measurement precision”. Coyle evaluated an inexpensive psychrometer for estimation of wet bulb temperature [33]. Omori commented on psychrometer, “Experiments have also been carried out to examine effects of atmospheric pressure, size of psychrometer, ventilation and conditions of super cooling or freezing of wet bulb, obtaining good agreement with the theory” [34]. Yin *et al.* introduced a parameterized psychrometer coefficient with wind speed in their paper [22].

From the above discussion and the literature, it is clear that the psychrometer has been widely used as a method for relative humidity measurement over a wide range of values in a harsh environment, with varying degrees of measurement accuracy. Therefore, it is important to study the interrelated elements in the measurement environment and optimize the measurement accuracy of the psychrometer. 

This paper introduces an improved measurement device of aspirating psychrometer with specific construction parameters and provided real test results that are conducted in a meat-drying room in a food company. A specific psychrometric measurement device was developed and tested, including the structure of the improved psychrometer, signal conversion method, calculating unit, and the real-time test results. The influences of the psychrometer structure and interrelated factors of the measurement environment were demonstrated. The research analyzed the accuracy, stability, and reliability of the specific improved psychrometric measurement method based on a real-time temperature and relative humidity process control system.

## 2. The Improvement

In this section, a common psychrometer implementation is briefly given. Then, a method of signal conversion between the dry and wet temperature probe measurements and relative humidity that is suitable for digital processors is presented.

### 2.1. Aspirated Psychrometer Device Structure

We tested several temperature sensors, including the thermocouple temperature sensor and the resistance thermometer. The PT100 is a resistance thermometer showed better accuracy and consistency than the thermocouple temperature sensors. Furthermore, the thermocouple temperature and the resistance temperature have a strong hull of stainless steel and its ingress protection number is 67. Thus, we selected the PT100 as the dry and wet temperature probes for the improved aspirated psychrometer. In order to fit the special measurement environment, the probes were chosen to be stainless steel with waterproof cable connections like IP67 ingress protection. The wet bulb temperature probe was soaked in water via a piece of cotton cloth. A fan was used to allow consistent and controllable airflow over the probes. The dry bulb and wet bulb temperature are denote by $T_{d}$ and $T_{w}$, respectively.

### 2.2. Algorithm for Psychrometer Signal Conversion

As an indirect measurement method, signal conversion between temperature and relative humidity is an important factor that directly affects the measurement accuracy of the psychrometer. Prior references exist on different conversion methods [35,36] and the software package referenced in Lv and Chen [37].

The signal conversion is based on the Sprung primary formula [38]. The mass of water vapour in a certain volume is defined as absolute humidity [1]. If ideal gas behaviour is assumed, the absolute humidity can be calculated by

(1) $$A=\frac{EP_{w}}{T}$$

The ratio of the partial pressure of water vapour in the mixture to the saturated pressure of water vapour at a prescribed temperature is defined as relative humidity, normally expressed as a percentage by

(2) $$RH=\frac{P_{d}\left(H_{2}O\right)}{P_{s}\left(H_{2}O\right)}\times 100\%$$

where $P_{d}\;$is the partial pressure of water vapour in the mixture in equilibrium and the water vapour pressure is a function of temperature. Dalton’s Law of partial pressures states that the total pressure of mixture of gases is the sum of the individual pressure gas in the mixture and it works only for ideal gases situation [39]. $P_{s}$ is the saturated pressure of water vapour at a prescribed temperature. 

The partial pressure of water vapour in the mixture can be calculated using the Sprung formula [38] by

(3) $$P_{d}=P_{w\;}–C\;\left(T_{d}-T_{w}\right)\frac{P}{755}$$

where *P* is the station pressure, $P_{w}\;$is the saturation pressure at wet bulb temperature, $T_{d}$ is the dry bulb temperature, $T_{w}$ is the wet bulb temperature, and *C* is a constant. The value of *C* differs according to the measurement environment. When the ambient temperature is above freezing, *C* = 0.5; and when the ambient temperature is below freezing, *C* = 0.43 [38]. In this study, the Sprung formula was tested to be valid when the temperature is the range of 4–18 °C in the tested meat drying processes. The range of Sprung psychrometric formula [40] is valid in the range of psychrometer chart (range −45–60 °C) [41]. In a NASA technical note, Parish and Putnam stated that the Sprung equations are valid when the ambient temperature range is at −50–100 °C [42]. The unit of temperature is degrees Celsius and the station pressure is in millbars/hectopascals. 

The $P_{s}$ and $P_{w}$ can be calculated by

(4) $$P_{s}=\;6.112\text{exp}\left(\frac{17.67T_{d}}{T_{d}\;+243.5}\right)$$

(5) $$P_{w}=\;6.112\text{exp}\left(\frac{17.67T_{w}}{T_{w}\;+243.5}\right)$$

These two equations are tested to valid for meat drying processes when the temperature is the in range of 4–18 °C. Finally, the relative humidity RH is obtained by Equation (2). 

### 2.3. Environmental Influences on the Psychrometer

Based on the measurement methods for relative humidity, the humidity sensors can be classified into two categories: direct and indirect measurement methods. The measurement principles of capacitive humidity sensor and resistive humidity sensor belong to the category of direct measurement methods. The indirect measurement methods of humidity do not expose the humidity sensitive device to the measurement environment. The psychrometric measurement method is a typical indirect relative humidity measurement method, which measure the temperatures and then convert them into RH values.

This research focuses on the structural parameter’s improvement of the aspirated psychrometer and specifically analyzes the influence of several factors in the measurement accuracy of the aspirated psychrometer based on the practical measurement. The experiments that were conducted included: the air flow speed test that air flow is passing through the dry and wet bulb temperature probes; the cloth size test that is put on the wet bulb probe; and the distance test between the wet bulb probe and water level. A specific improved psychrometer device was implemented in order to perform these experiments. 

**Figure 2:**
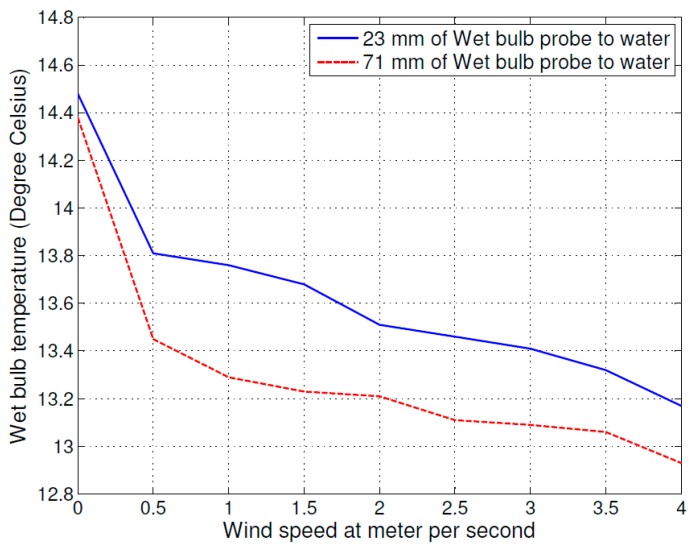
The relation between the air flow speed and wet bulb temperature (cloth size: 63 mm × 122 mm).

There are different styles of psychrometers, and for the same style of psychrometer, the construction sizes are mostly different. The motivation to select two different distances to demonstrate that the effect of the distance between the water level and the wet bulb cannot be ignored. The first experiment was set up with two different distances between the wet bulb probe and the water level, at 23 mm and 71 mm. The variable was the air flow speed, which was changed from 0 m/s (stationary) to 4 m/s at 0.5 m/s intervals. The size of the wet cloth was 63 mm × 122 mm. The test results are shown in Figure 2, where the solid line curve shows the 23 mm setup and the dashed line curve shows the 71 mm setup. The wet bulb temperature probe with a greater distance from the water level shows a lower measured temperature because the larger distance increased the rate of evaporation off of the cloth. From left to right, the higher velocity of air flow also increased the evaporation process and decreased the temperature of the wet bulb.

**Figure 3:**
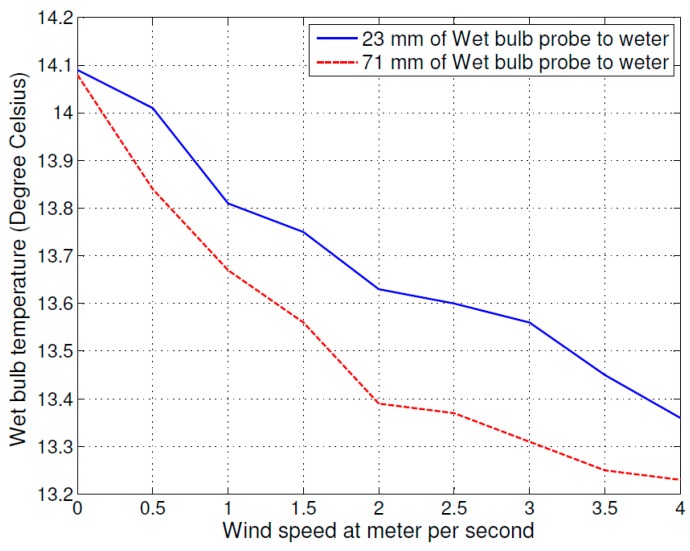
The relation of the air flow speed with wet bulb temperature (cloth size: 126 mm × 122 mm).

The second experiment was designed to examine the influence of different cloth size to wet bulb temperature. In the experiment, the cloth size was changed to 126 mm × 122 mm and other variables were same as in the first experiment. The test result is shown in Figure 3, where the solid line curve shows the 23 mm setup and the dashed line curve shows the 71 mm setup. The experiment result shows, the evaporation process of small cloth size is faster than big cloth size. Again, from left to right, the higher air flow speed increased evaporation process and decreases the temperature of wet bulb.

The influence of environmental factors is shown in Figure 2 and Figure 3, which include the air flow speed distance between wet bulb and water level, and wet bulb cloth size on the psychrometer. A larger distance between the wet bulb and water level increased the evaporating process and decreased the wet bulb temperature. The higher air flow speed increased the evaporating process and decreased the wet bulb temperature. Lastly, comparing the curves in Figure 2 and Figure 3, the different cloth sizes also caused the wet bulb temperature change. A smaller cloth size caused the temperature of wet bulb to decrease faster.

Based on the above results, we estimated the sensitivity of wet bulb temperature to the variation of various environmental factors. At a constant air flow speed of 2 m/s, different cloth sizes caused 0.2 °C uncertainty in the wet bulb temperature. Different distances between wet bulb temperature probe to water level added 0.3 °C uncertainty, and if the air flow speed changed from 2 m/s to 0.5 m/s then the uncertainties would increase approximately 0.3 °C. The total change was approximately 0.8 °C of the measured wet bulb temperature value.

## 3. Results and Discussion

From Section 3, it is clear that the measurement accuracy of the psychrometer can be improved with appropriate adjustments of various factors. 

Based on the analysis in Section 3, an improved psychrometer measurement device was developed with several key factors chosen to improve measurement accuracy. These factors included the distance between the dry and wet bulb probes (72 mm), wet cloth size (127 mm × 125 mm), the distance between the wet bulb and water (43 mm), the distance from blow fan to dry bulb and wet bulb probe (66 mm), the air direction from fan to wet bulb probe and the air flow speed (5.25–5.3 m/s), the accuracy of resistance temperature detectors, and signal conversion. We have conducted tests using different measurement devices, such as process control instrument, Jumo lpf100, and Siemens PLC step7-200. We got good measurement results using all devices using the construction parameters presented in this paper.

The dry bulb and wet bulb temperature signals were measured in a three-wire circuit using a specific integrated circuit-ASIC 7030 A/D for analog to digital conversion. The profile controller operated with a micro-controller to acquire the measurement data. The test instruments include A/D converter and micro controller. The ASIC 7030 was used as the A/D converter. The two styles of micro controllers used were Neuron chip 3150 BF by Toshiba Corporation Semiconductor Company (Tokyo, Japan) and 89 C52 by ATMEL Ltd. (1600 Technology Drive, San Jose, CA, USA). Several accurate instruments were used to calibrate the specific improved psychrometer measurement device and measure its accuracy. The instruments included: VAISALA humidity and temperature probe (HMP75) and measurement indicator (MI70), PACER digital anemometer (DA400), Ebro precision thermometer (TFX 430), MadgeTech accurate data logger (RHTemp 101A) for temperature and relative humidity, and Fluke instrument dry-well (9102S).

In these experiments, the dry-well (Fluke 9102S) and Ebro precision thermometer (TFX 430) were used for dry bulb and wet bulb temperature probe calibration. The accuracy of Ebro precision thermometer (TFX 430) is ±0.05 °C and resolution is 0.01 °C. The humidity and temperature probe (HMP75) and measurement indicator (MI70) were used as a confirmation instrument. Table 1 shows the calibration results of humidity and temperature for Probe HMP75 by Vaisala Oyj, Helsinki, Finland.

### 3.1. Measurement Accuracy of Improved Psychrometer

**Figure 4:**
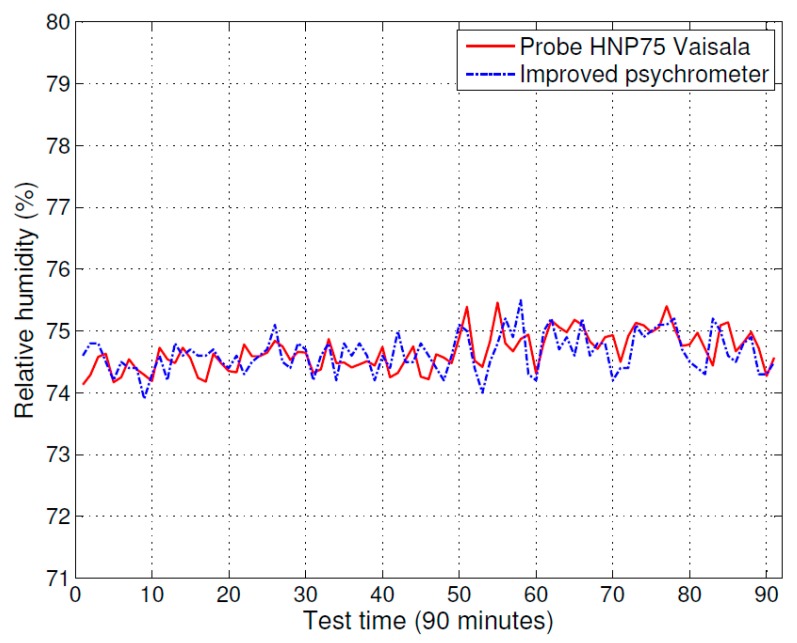
Comparison of measurement accuracy between the improved psychrometer and relative humidity instrument (HMP75).

**Figure 5:**
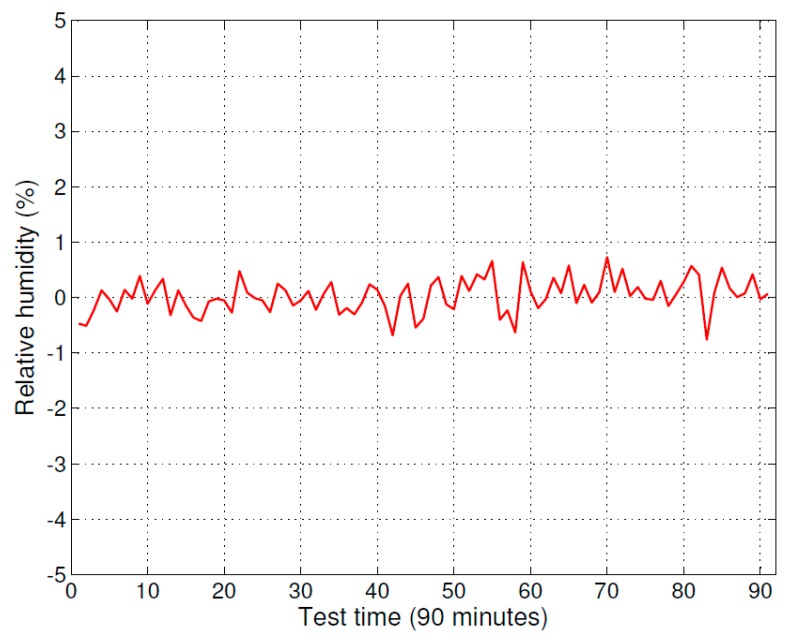
Difference of measurement value between the improved psychrometer and comparison instrument (HMP75).

The improved psychrometer and the humidity and temperature probe (HMP75) with measurement indicator (MI70) were tested in a drying room environment. The test results of the improved psychrometer and the humidity measurement instrument (MI70 with HMP75) are shown in Figure 4. The solid line curve indicates the measurement result of the humidity measurement instrument (MI70 with HMP75) and the dot-dashed line curve indicates the measurement result of the improved psychrometer. The difference between the measurements results, specifically the improved psychrometer measurement value minus the measurement value of humidity measurement instrument (MI70 with HMP75), are shown in Figure 5.

The test data shows that the measurement value of the improved psychrometer is very close to the measurement value of the humidity measurement instrument (MI70 with HMP75). The deviation between the two instruments was less than ±0.5% RH. According to the certified accuracy of the humidity measurement instrument (MI70 with HMP75), the measurement accuracy of the improved psychrometer was ±0.6% RH in a drying room measurement environment.

### 3.2. Comparison of Original Psychrometer and Improved Psychrometer

A comparison test was made between the original psychrometer and the improved psychrometer in a functioning meat drying room control environment. The original psychrometer device was removed from the same general drying room control system.

The original psychrometer device and the improved psychrometer device used the same probe and instrument. The calibration instrument was the same as in the previous section, and the atmosphere pressure was 964 hPa at the time of the experiment.

**Figure 6:**
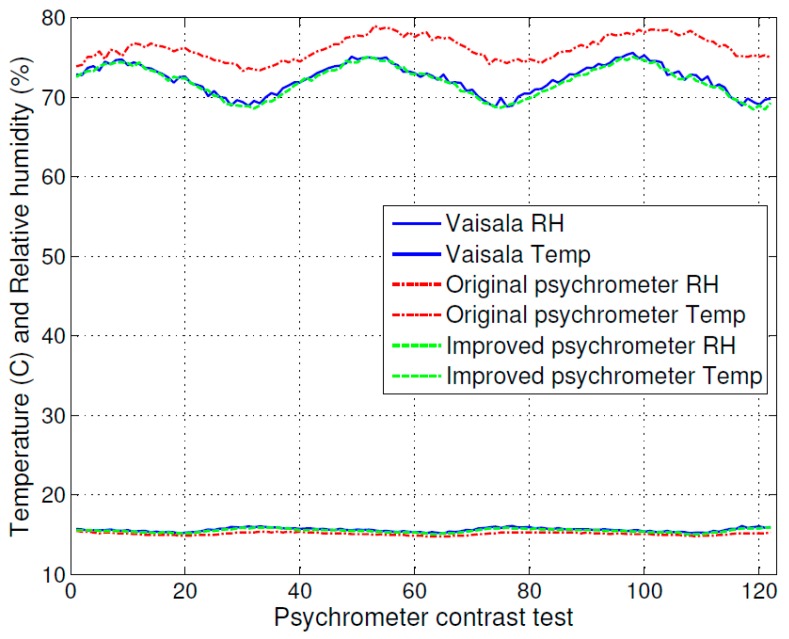
Comparison of temperature and relative humidity using the original psychrometer, the improved psychrometer, and the relative humidity measurement instrument (HMP75).

**Figure 7:**
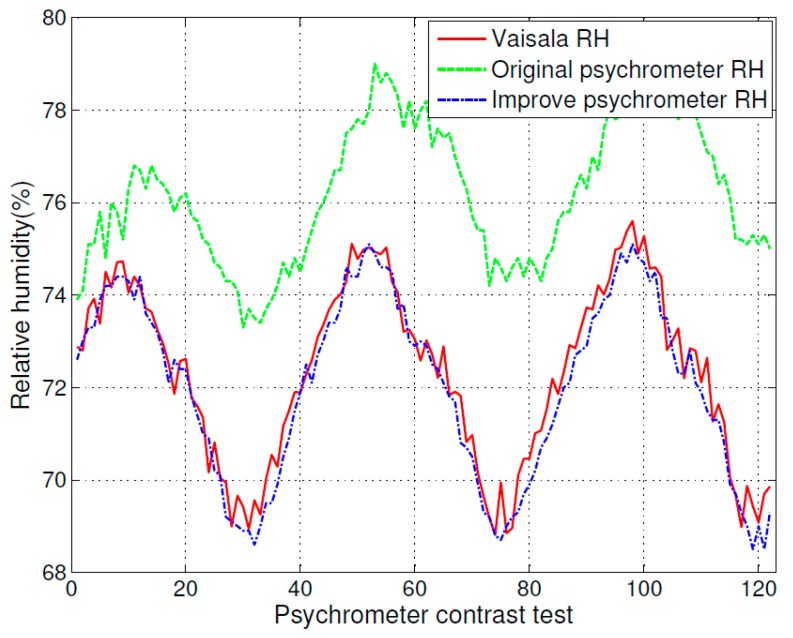
Comparison of relative humidity using the original psychrometer, the improved psychrometer, and the measurement instrument (HMP75), a zoomed part of Figure 6.

The test results for temperature and relative humidity are shown in Figure 6. Figure 7 is an extract of Figure 6 that shows the detailed test result for relative humidity. At the top of Figure 6, the dot-dashed line curve indicates the relative humidity measurement result of the humidity measurement instrument (MI70 with HMP75), the solid line curve indicates the relative humidity measurement result of the original psychrometer, and the dashed line curve indicates the relative humidity measurement result of the improved psychrometer. At the bottom of Figure 6, the dot-dashed line curve indicates the temperature measurement result of the humidity measurement instrument (MI70 with HMP75), the dot line curve indicates the temperature measurement result of the original psychrometer, and the plus sign line curve indicates the temperature measurement result of the improved psychrometer.

The measurement accuracy of the original psychrometer and the improved psychrometer are clearly shown in Figure 7. The relative humidity measurement curve of the improved psychrometer is very close to the measurement curve of the calibrated humidity and temperature probe (HMP75) with the measurement indicator (MI70), and the test result shows that the original psychrometer has more error.

### 3.3. Stability Analysis of the Improved Psychrometer

Another issue of a relative humidity sensor is the stability of measurement signal. Most relative humidity sensors have zero drift issue which depends on the properties of the relative humidity sensitive element. This issue is more evident in a harsh environment such as the drying room for meat production. 

Measurement stability of the prototype psychrometer was quantitatively analyzed in a real-time temperature and relative humidity control system. The experiments in a drying room control system using the prototype psychrometer as the relative humidity sensor was conducted. The testing environment was challenging since the air contained smoke particles, which did not allow capacitive relative humidity sensors to work at any extended period of time. It is a big challenge to have a sensor work in meat-drying room environments to measure the relative humidity. Since the humidity sensitive device of capacitive humidity sensors or resistance humidity sensors is exposed in a measurement area, it cannot perform properly in a harsh environment. The real tests show that some relative humidity sensors, such as capacitive humidity sensors or resistance humidity sensor, do not work properly for a long period of a time. A psychrometer does not use the humidity sensitive device for relative humidity measurement. It uses two temperature probes, named the dry bulb and wet bulb probes, to measure the relative humidity indirectly. This feature of measurement demonstrates that the modified psychrometer cam work stably in a harsh measurement environment for a very long period of time.

The dimension of the test drying room was 28 m × 9 m × 3.8 m. The control variables were the room temperature and relative humidity. The cooling source of the system was NH_3_ at −7 °C, and heating source of the system was low pressure steam at 15 *PSI*. The humidifier was a dry fog generator with water droplet size of ≤4.2 microns. The drying room control system was two independent closed-loop control systems for temperature and relative humidity using the proportional, integral, and differential control algorithm. The relative humidity sensor was a prototype psychrometer.

Two temperature and relative humidity data loggers (MadgeTech TempRetriever-RH and RHTemp101A) were used as the recording measurement equipment. Among them, the TempRetriever-RH had a relative humidity resolution of 0.1% RH and a temperature resolution of 0.1 °C. The RHTemp101A had a relative humidity resolution of 0.1% RH and a temperature resolution of 0.01 °C. 

**Figure 8:**
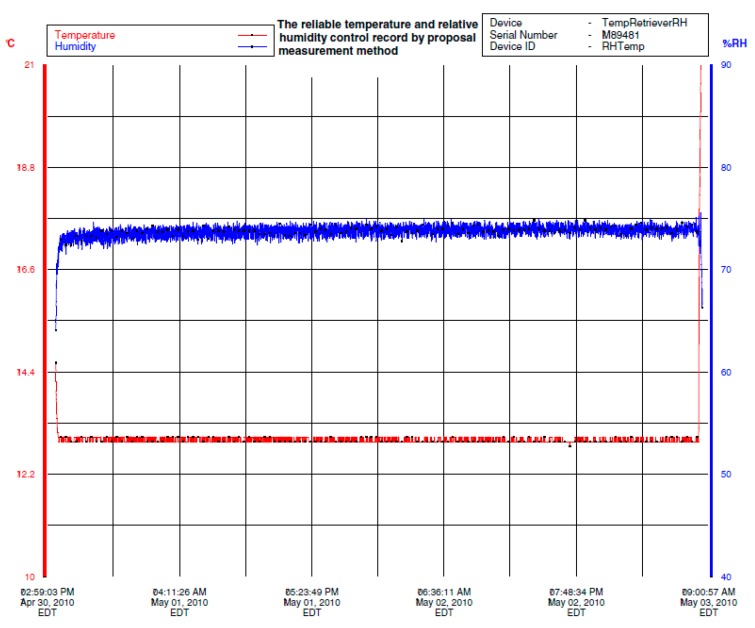
The process control results of temperature and relative humidity based on the improved psychrometer.

The set points of temperature and relative humidity for the drying room control system were 13 °C and 74% RH. The test results of temperature and relative humidity under closed-loop control are shown in Figure 8. The bottom curve of left vertical axis indicates temperature values in Celsius degree (°C) and the top curve of right vertical axis indicates relative humidity values in percentage (%). 

**Figure 9:**
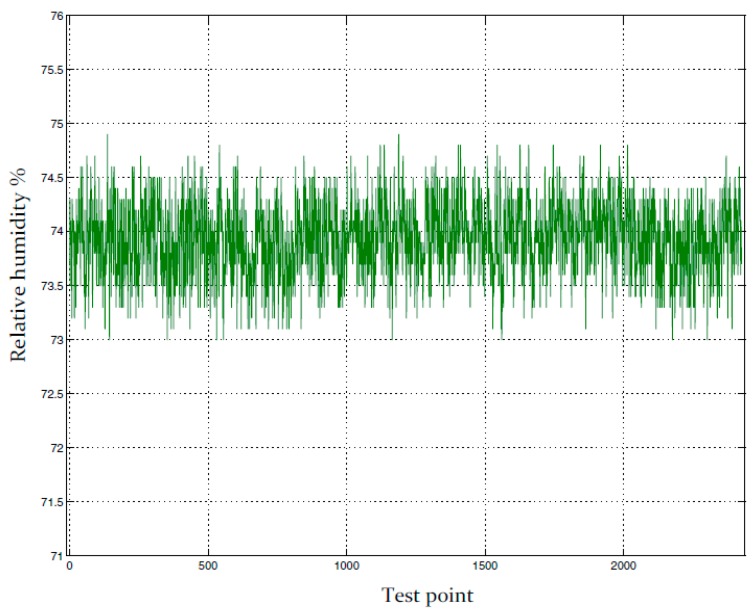
The accuracy analysis of relative humidity process control based on the improved psychrometer.

The 65 h recording of temperature and relative humidity in a drying room under closed-loop control is shown in Figure 8 where the prototype psychrometer device was used as the relative humidity sensor. Figure 9 is part of Figure 8 and shows a small portion of the relative humidity recording. Based on Figure 9, the relative humidity control accuracy was within ±0.8% RH. The test results clearly show the prototype psychrometer device can provide stable measurements in a tough measurement environment for an extended period of time and can provide higher relative humidity control accuracy in an industrial meat drying room.

### 3.4. The Influence of Temperature in the Measurement Accuracy of Relative Humidity

Temperature directly influences relative humidity measurement accuracy and stability. The effect of the varying factors in the relative humidity measurement can be evaluated using Table 2. The coupling relationship of temperature and relative humidity close to the values of 13 °C and 73% RH are given in Table 2. Table 2 shows the coupling relationship of temperature and relative humidity in a hermetically sealed container. 

**Figure 10:**
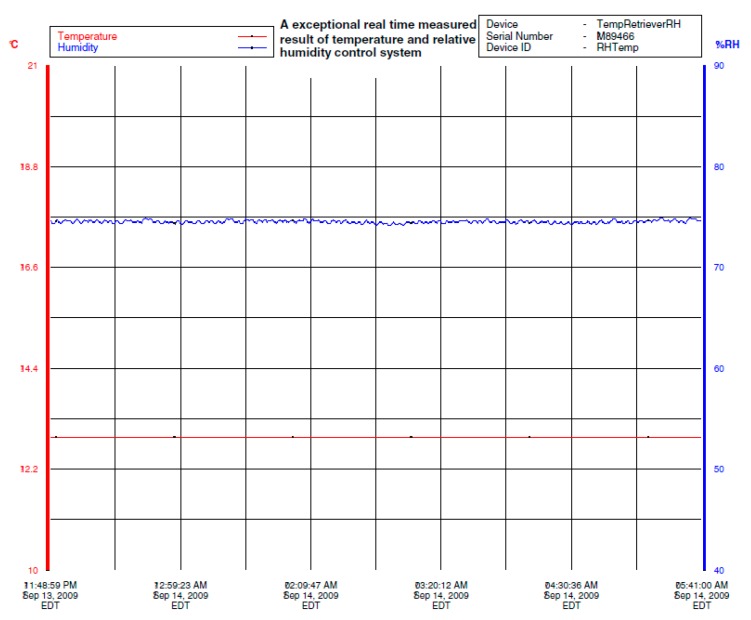
The temperature and relative humidity under closed loop control based on meticulous setup.

**Figure 11:**
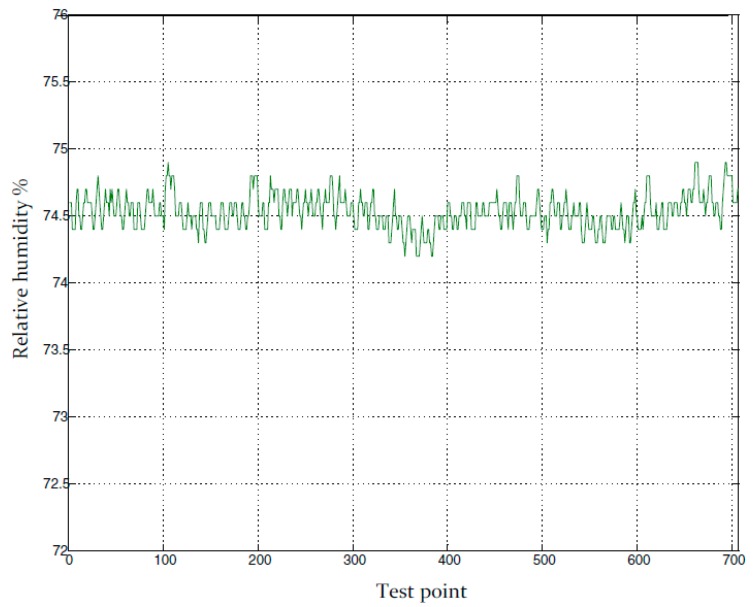
The relative humidity under closed loop control with temperature fluctuation ±0.05 °C.

Based on Table 2, the temperature change of ±1 °C will lead to the relative humidity change at least 4% RH. The values of the temperature fluctuation is ±0.1 °C in Figure 8, taking into account the instrument measurement uncertainty, the maximum temperature fluctuation is expected to be ≤±0.15 °C. The coupling influence of temperature to relative humidity is ±0.6% RH in Figure 9. Thus, because the influence of the coupling, the temperature control effect is directly affects the control accuracy of the relative humidity control system. The Figure 10 and Figure 11 illustrate the effect. 

The test results showed the temperature control accuracy was achieved ±0.05 °C for over seven hours in the control system. In Figure 10, the bottom curve indicates the temperature values in Celsius degree (°C) and the top curve indicates the relative humidity values in percentage (%). The control accuracy of relative humidity is shown in Figure 11, which is a zoomed-in version of Figure 10. The relative humidity control accuracy was ±0.5% RH for the drying room control system using a prototype psychrometer as the relative humidity sensor.

The above test results distinctly revealed the influence of temperature control accuracy to the relative humidity control accuracy in the drying room control system. When the temperature control accuracy was ±0.1 °C, the relative humidity control accuracy was ±0.8% RH (Figure 9), and when the temperature control accuracy reached ±0.05 °C, the relative humidity control accuracy was reached ±0.5% RH (Figure 11).

## 4. Conclusions

This paper focused on a discussion of the application for the relative humidity measurement device in a current control system in challenging environments, in particular, the drying system for dry-cured meat products. Test results revealed the effects of different parameters of the psychrometer in the measurement accuracy and stability of relative humidity.

Based on experimental results, the characteristics of the specific improved psychrometer are summarized as follows.

1. The relative humidity measurement accuracy of the specific improved psychrometer that can be achieved is ±0.6% RH in a meat manufacturing environment.
2. The specific improved psychrometer can perform long-term, stable, and reliable work in a severe environment of a meat manufacturing drying room.
3. Based on the test results of a drying system for dry-cured meat products using a specific improved psychrometer, the control accuracy of a relative humidity system that can be achieved is ±0.8% RH.

In summary, these characteristics of the improved psychrometer, including its higher measurement accuracy and stability, make the psychrometer especially suitable as a relative humidity sensor for applications in challenging measurement environments.

## Figures and Tables

**Table 1:** Calibration Results of the Humidity and Temperature for Probe HMP75.

Humidity/Temperature	Measured Value 1 Unit	Measured Value 2 Unit	Measured Value 3 Unit
Reference relative humidity	+33.1% RH	+54.0% RH	+74.7% RH
Reference temperature	+22.06 °C	+22.05 °C	+22.05 °C
Observed relative humidity	+33.7% RH	+54.5% RH	+74.9% RH
Observed temperature	+22.06 °C	+22.04 °C	+22.05 °C
Relative humidity difference	+0.6% RH	+0.5% RH	+0.2% RH
Permissible relative humidity difference	±1.0% RH	±1.0% RH	±1.0% RH

**Table 2:** The coupling relation of temperature and relative humidity at the proximity of 13 °C and 73% RH.

Humidity/Temperature	Unit								
	1	2	3	4	5	6	7	8	9
Temperature (°C)	11	11.5	12	12.5	13	13.5	14	14.5	15
Relative humidity (% RH)	83.3	80.6	78	75.4	73	70.7	68.4	66.2	64.1
